# The Height of Antisepsis

**Published:** 1910-12-31

**Authors:** C. O. Hawthorne

**Affiliations:** Harley Street, W., December 26, 1910.


*To the Editor of* The Hospital.

### THE HEIGHT OF ANTISEPSIS.

*To the Editor of* The Hospital.

Sir,—The amusing story (quoted in your columns from *Le Petit Moniteur de la Médecine*) of the surgeon, who, assisting at a duel, insisted on a preliminary immersion of the rapiers in carbolic solution, has a parallel or precedent in an incident related by Mr. Ellis Roberts in his recently published *Samuel Rogers and His Circle*. The hero again was a surgeon, but was one of the principals, not merely an assistant, in the duly arranged duel, and he was certainly not less logical and not less thorough than our French *confrère*. The record runs as follows : "Humphrey Howarth, the surgeon, was called out, and made his appearance in the field stark naked, to the astonishment of the challenger, who asked him what he meant. 'I know,' said Howarth, 'that if any part of the clothing is carried into the body by a gunshot wound festering ensues; and therefore I have met you thus.' His antagonist replied that fighting with a man *in puris naturalibus* would be quite ridiculous; and accordingly they parted without further discussion." The date of this affair is not quoted, but as it must have been at least a century ago, Mr. Howarth seems to be entitled to the credit of a very early, possibly the earliest, appreciation of the advantages of aseptic surgery. Hence if the French surgeon may be distinguished as the *anti*septic surgeon *in excelsis*, his British forerunner surely appears as a shining example of early and complete *a*sepsis. The logic of the situation must not be monopolised by our brethren across the Channel.—I am, sir, yours faithfully,

C. O. Hawthorne.

Harley Street, W., December 26, 1910.

